# New perspectives on the evolution of women's cooperation

**DOI:** 10.1098/rstb.2021.0424

**Published:** 2023-01-16

**Authors:** Stephanie A. Fox, Brooke Scelza, Joan Silk, Karen L. Kramer

**Affiliations:** ^1^ Department of Anthropology, University of New Mexico, Albuquerque, NM 87131, USA; ^2^ Department of Anthropology, UCLA, Los Angeles, CA 90095, USA; ^3^ School of Human Evolution and Social Change, Arizona State University, Tempe, AZ 85281, USA; ^4^ Department of Anthropology, University of Utah, Salt Lake City, UT 84112, USA

**Keywords:** women, cooperation, evolution, sex differences, prosociality

## Abstract

A holistic, evolutionary framework about human cooperation must incorporate information about women's cooperative behaviour. Yet, most empirical research on human cooperation has centered on men's behaviour or been derived from experimental studies conducted in western, industrialized populations. These bodies of data are unlikely to accurately represent human behavioural diversity. To address this gap and provide a more balanced view of human cooperation, this issue presents substantial new data and multi-disciplinary perspectives to document the complexity of women's cooperative behaviour. Research in this issue 1) challenges narratives about universal gender differences in cooperation, 2) reconsiders patrilocality and access to kin as constraints on women's cooperation, 3) reviews evidence for a connection between social support and women's health and 4) examines the phylogenetic roots of female cooperation. Here, we discuss the steps taken in this issue toward a more complete and evidence-based understanding of the role that cooperation plays in women's and girls' lives and in building human sociality.

This article is part of the theme issue ‘Cooperation among women: evolutionary and cross-cultural perspectives’.

## 1. Introduction

Over the past several million years, humans have become a globally dominant species by greatly expanding the scale at which we participate in cooperative activities and leveraging our capacity for cumulative culture [1,2]. Cooperation is a central and universal element in human societies and foundational to derived human traits, such as food sharing, pooled energy budgets and the division of labour [3–5]. There is also a growing consensus that cooperation is critical to sustaining elements of human life history and cognition [6–9]. Much attention has been paid to the cooperative childrearing networks required to buffer the costs human mothers face in caring for multiple dependent offspring at the same time [10–14]. The breadth of a mother's cooperative childcare network, composed of her children, female relatives, other kin and nonkin, is well documented across a number of societies [15–21], as is women's cooperative food production [22–27]. However, much less attention has been paid to the many ways that women and girls engage in cooperative political, ceremonial, economic and social institutions, collectively form coalitions and develop exchange and support networks. Women's cooperative activities and roles across these domains are likely important cohesive forces in building human societies and maintaining intergroup relations [28–32]. Yet, empirical syntheses, quantitative investigations and theoretic development on this topic remain limited. Consequently, women's cooperation has been neglected in reconstructions of the evolution of human sociality [4,33–36]. This special issue urges a more balanced understanding of gender differences in the propensity to cooperate and the evolutionary processes that shaped human society. In this introduction, we review the theoretical backdrop for this theme issue and synthesize ways in which contributions in the issue illuminate the diversity and complexity of women's cooperation.

Theoretical models and empirical analyses of cooperation's role in human societies have often focused on male-dominated domains, particularly hunting, warfare and leadership. Researchers have emphasized the importance of men's cooperation in hunting because returns from hunting are often highly variable and sharing among hunters reduces variance in food acquisition [8,37,38]. Others have emphasized men's inclination to cooperate in large groups, even when individuals have relatively shallow relationships, to protect territory and engage in intergroup warfare [39–42]. Hunting, inter-group defense and coalitionary political support have all been argued to favour male alliances and cooperation and are considered less integral to how women organize [39,42–51]. Emphasis on men's cooperation also stems from common but unsupported expectations about the prevalence of male philopatry and patrilocality during human evolution ([47,52,53]; but see [54]). The assumed centrality of male bonding has overemphasized women's isolation from their kin; competition with other women for mating opportunities, allocare and resources; and men's primacy to band together, and has led some scientists to theorize about biological sex differences in male and female predispositions to cooperate [42,46,55,56]. Further supporting this perspective, some experimental psychology studies have emphasized gender differences in the propensity to cooperate, suggesting that men's and boys’ same-sex relationships are more cooperative and that they tend to cooperate in larger groups [43,45,53,55–66]. However, the majority of these experiments were conducted in western, industrialized populations. Collectively, these biases in current research are unlikely to accurately represent the full range of human behavioural diversity. In this issue, we approach the male-dominated narrative through a critical lens and ask whether gender differences in cooperation are supported when women's behaviour is examined from a broader, developmental and cross-cultural perspective.

This issue brings together research from behavioural ecology, anthropology, health psychology, developmental psychology and behavioural economics to construct a more complete view of the roles that cooperation and competition play in the lives of women and girls. Papers explore the phylogenetic legacy of female cooperation by considering sex differences in cooperation among mammals and the nature of female relationships in our close living relatives, chimpanzees and gorillas. Several ethnographic case studies highlight the breadth of women's cooperative activities, how women structure their social networks and the range of contextual factors that influence the extent of female cooperation, including relatedness, inheritance systems and social norms. Contributions also assess experimental evidence for gender differences in prosocial behaviour during development and adulthood and review the relationship between female social ties and physical, psychological and relationship health.

Taken together, the research in this issue does not support universal gender differences in cooperative tendencies. Rather, the evidence points to the important function that cooperation plays in the lives of women, who build multifaceted cooperative relationships across many domains and ages, and in a range of household, community and intergroup settings.

## 2. A note about terms

This issue focuses on topics linked to sex and gender across nonhuman and human species, from biological and cultural perspectives. Though we are hypothesizing about the evolution of behaviours potentially linked to sex, we are discussing gendered experiences in humans; thus, we generally use the terms *women*, *girls*, *men* and *boys* when referring to humans, and use *female* and *male* when making cross-species comparisons or discussing reproductive biology. However, the use of these terms is nuanced. Authors use sex or gender terms according to what is most appropriate for their human participants. Additionally, we expect some variation across studies in the extent to which participants identify with sex or gender terms given that they have different meanings across cultures. Addressing the full spectrum of gendered experiences in cooperation is important complementary research but goes beyond the scope of the present issue.

## 3. Key themes and findings from this issue

Several themes and findings emerge from the special issue and highlight the ways in which female cooperation needs to be reconsidered.

### (a) No compelling evidence for universal gender differences in cooperative networks or prosocial behaviour

Sexual selection theory has been extended to make predictions about how men and women may use cooperative networks differently [41,42,46,47,53,55,56,67,68]. In brief, among mammals, male reproduction is generally limited by access to mates. This, combined with the pace of human life history, favours male engagement in high-risk strategies, mate guarding and pursuit of high social status. By extension, men and boys are expected to form political alliances and engage in collective mate defense through intergroup aggression, and benefit from forming broad, diffuse social networks. On the other hand, the reproductive success of mammalian females is generally limited by access to resources. As a consequence, women are expected to engage in low-risk strategies that enhance their own health and survival and that of their children. Women and girls are expected to build ties that are linked to childcare, be highly selective in choosing cooperative partners, form narrow social networks, exclude competitors and cooperate mainly with close kin.

Evidence for this theoretical framework has been mixed. In support, several studies characterize women and girls as forming fewer same-sex friendships, investing more in each relationship, being more likely to terminate relationships over social transgressions and being less cooperative than men and boys in same-sex interactions [43,45,53,55–61,63–66]. On the other hand, research from social and health psychology indicates that women are more likely to respond to stressful scenarios by seeking and providing support and engaging in other-oriented strategies despite interacting with strangers, whereas men are more likely to respond selfishly and competitively ([69–72], reviewed in [73]). Further, a review on children's prosocial behaviour found that girls were often found to have smaller playgroups, but they were more prosocial than boys in most other respects [64]. However, these studies have mainly been conducted with children and undergraduate students in western, educated, industrial, religious, democratic (WEIRD) societies [74–76].

The ethnographic record is also mixed. One study comparing ethnographic records from small-scale societies argued that men often cooperate in larger groups while women are more likely to engage in non-cooperative, parallel activities [42]. By contrast, in a study with Hadza hunter–gatherers in Tanzania, men and women did not differ in their number of preferred campmates or in the number of individuals with whom they would share gifts [77]. An absence of gender differences in social networks has also been found in other studies [78–80]. These findings are consistent with two studies in this issue that found no substantial gender differences in cooperative networks [81,82]. In detailed studies of two Tamil villages (South Indian mixed economy), Simpson and Power [82] showed that men and women use somewhat different strategies to obtain help, but these differences were statistically modest and did not generate substantive gender differences in the structure of support networks. Similarly, in a study of matrilineal and patrilineal Mosuo communities (Tibetan-descended agriculturalists), Mattison and colleagues [81] found that differences in men's and women's cooperative networks did not follow expected gender patterns.

The ethnographic evidence of relatively subtle and inconsistent differences in network characteristics in small-scale societies is reflected in experimental games that assess prosociality and cooperation across cultures. In this issue, House and colleagues [83] found no evidence for gender differences in prosociality and fairness among children in 21 diverse societies. Complementing this developmental study, Spadaro and colleagues ([84], in this issue) found no consistent gender differences in experiments designed to evaluate cooperative tendencies among adults in 20 industrialized societies, in contrast to a previous but less diverse meta-analysis [57]. Echoing Hruschka [85], the papers in this special issue indicate that claims about universal gender differences in cooperation are overstated. Given the range of findings across studies, we suggest that further investigation into the situational and societal contexts that intensify or diminish cooperative gender differences will be more fruitful than searching for universal differences.

### (b) Women's cooperation extends beyond kinship lines

As with other female mammals, cooperation among women and girls often falls along kinship lines [20,86–89]. Importantly, though, the notion that access to kin *limits* female cooperation contradicts the empirical record and needs to be reconsidered.

Discussions of cooperation among women often highlight the consequences of postmarital residence, with patrilocal residence expected to limit women's access to cooperative partners [42,47,52,53]. However, longitudinal ethnographic research has shown that residence patterns are more flexible than previously realized [90–93]. Even where patrilocality is prevalent, women develop workarounds that enable them to find new partners or maintain connections with natal kin [54,94–97]. In an analysis that compared supportive relationships among women in rural Bangladesh who emigrated to their husband's village and those who stayed in their natal village when they married, Hruschka and colleagues ([97], in this issue) found that patrilocality did not isolate women. Women who migrated from their natal community initially lived in proximity to few kin, but they quickly built social support relations with close affines and friends. In a similar finding, the social group size of Tsimane women (South American horticulturalists) did not differ between women in different post-marital residence settings when they engaged in activities such as gardening and wage labour, manufacturing and resource acquisition. However, the distance a woman lived from her parents, regardless of whether she lived patrilocally, matrilocally or neolocally, influenced her social group size and her probability of receiving allocare ([98], in this issue). Likewise, among the patrilineal Mosuo, women's food preparation networks were better predicted by geographical distance than by genetic relatedness ([81], in this issue). Finally, Simpson & Power ([82], in this issue) assessed help-seeking among patrilocal Tamil men and women and found no evidence that women's social network ties are limited to kin. Taken as a whole, findings from this issue challenge the conventional assumption that patrilocality constrains women's cooperation and urge us to consider the importance of affinal kin and friendships in women's cooperation.

Women's capacity to flexibly form cooperative ties both with kin and non-kin may stem from a long evolutionary history of navigating relationships in contexts with differential access to kin. Supporting this idea, research on female great apes demonstrates that between-species variation in access to kin does not easily predict levels of female cooperation. Though great ape social systems vary widely, females in all species typically disperse from their natal groups and it is uncommon for adult females to live with female kin [99]. Regardless, in some species females form well-differentiated relationships and cooperate with one another. At one end of the spectrum, bonobo females are highly social and cooperative with each other, despite living in a male philopatric social system, and even engage in friendly interactions with females from neighbouring groups [100–103]. Female chimpanzees are less social than bonobos, but engage in selective, socially tolerant relationships that support cooperative, agonistic coalitions ([104], in this issue). Occasionally strong, kin-based relationships occur when female chimpanzees remain in their natal community as adults, yet neither strong bonds nor kinship bolster cooperation ([104], in this issue). In both western and mountain gorillas, females form differentiated, stable partner preferences, despite natal and secondary dispersal occurring regularly and limiting both access to kin and long-term investment in relationships ([105], in this issue). Female orangutans express yet another unique ape pattern, as they most often range only with offspring, but have some access to kin because their ranges frequently overlap with other female relatives [106–109]. Despite occasional access to kin and low rates of affiliation, kinship still biases social tolerance among adult female orangutans [110].

Further substantiating the argument that access to kin does not sufficiently explain variation in cooperation, Smith and colleagues ([111], in this issue) found no phylogenetic signal for sex differences in intragroup coalition formation across mammals. They also found no support for the prediction that in female philopatric species, females are more likely to exhibit intragroup coalitions than females in other species. Collectively, studies in humans, non-human apes and across mammals support the proposition that female cooperation extends beyond kin. Females may develop cooperative bonds regardless of where they reside after maturity, such that proximity to kin alone is an insufficient predictor of female social relationships.

### (c) Variation in women's cooperation reflects need, risk and cultural norms

Post-marital residence is only one facet of the socioecological context that affects women's cooperation. Kramer ([112], in this issue) argues that women's cooperation is responsive to a variety of factors, including cultural norms, life-history stage, subsistence strategy and household demography. In a similar vein, research in experimental settings indicates that women's cooperative behaviour may be more sensitive to their social partners' needs and behaviour than men's, and women may be more likely to shift social strategies depending on the costs and benefits of each scenario compared to men ([73,82,113], all in this issue).

In this issue, authors tested several novel predictions about how women's cooperation is influenced by socioecological context. For example, Page and colleagues ([114], in this issue) hypothesized that women's childcare networks respond to major livelihood transitions. They predicted that in the shift from mobile to sedentary residence, childcare networks should decrease in size since wealth accumulates with sedentarism, reducing the need to rely on large networks of cooperators. However, they did not find this to be the case for Agta women (Philippine foragers); rather, mothers had large and diverse childcare networks across both mobile and sedentary communities. In a cross-cultural study, Kraft and colleagues ([115], in this issue) found support for the prediction that the size and composition of cooperative food networks vary with the risks associated with food acquisition strategies. Their comparison of Batek (Malaysian hunter–gatherers) and Tsimane (Bolivian horticulturalists) showed that Batak women, who experienced greater variability in daily foraging success, had more diffuse and diverse networks in contrast to Tsimane women, who relied on fewer, dependable cooperative partners, who were most often close kin, for horticultural labour. As another perspective, Bedrov & Gable ([73], in this issue) proposed that cross-cultural differences in cooperation should vary with society-level individualism versus collectivism, as women seek and benefit from cooperation differently in these contexts. Together, these studies highlight that women's cooperation is responsive to socioecological context and local cultural norms. The continued development of new hypotheses will be required to produce a holistic perspective on the evolution of cooperation in humans that accounts for nuance and variation in women's cooperative strategies, as has been done with men's cooperation.

### (d) Cooperation and competition are intimately interconnected

Competition and cooperation are closely linked social strategies. The behavioural ecology literature provides many examples of males and females in group-living species using cooperative tactics to offset individual differences in rank and power and alter the outcome of intragroup conflicts [89,111,116,117]. The connection between competition and cooperation also emerges in research on women. For example, several researchers have proposed that women cooperatively engage in subtle, non-confrontational tactics, such as gossip and exclusion of others, to compete for status or mates [52,118–120]. In this issue, Cassar and Rigdon [113] hypothesize that women may suppress competitive behaviours in an effort to maintain the potential for cooperative relationships. In their overview, they highlight studies showing that girls frequently prefer cooperative over competitive games and are less tolerant of social status differences among peers than boys. The authors suggest that this is why women are less competitive in ‘winner takes all’ experimental games, but more competitive when experiments have the option for winners to share with losers.

In real-world scenarios, women's inclination to cooperate or compete can directly influence their reproductive fitness. In human societies, women depend on others for help in cooperatively raising offspring, and if multiple women rely on the same pool of helpers, it raises the potential for competition over access to help (reviewed in [121], in this issue). Most empirical studies of reproductive conflict have been conducted in patrilocal–patrilineal societies and among agro-pastoralists, and the findings have been mixed. Hackman & Kramer ([121], in this issue) assessed potential fitness effects of reproductive conflict in a group of Savanna Pumé women (South American hunter–gatherers) who typically live in their natal camps after marriage and rely on a common pool of helpers. Longitudinal data showed that the number of women living in the same camp, whether they are kin or non-kin, did not negatively affect women's fertility or their children's survivorship. This study points to the many ways that residential mobility and bilateral kin access reassort women to labour needs and resource constraints, readjust tensions and reduce the potential for reproductive conflict.

Research on women's cooperation will benefit from further considering how cooperation intersects with competition. Given that women's competition is expected to change in different contexts [54,113,120], and that women can use cooperation to compete, extending hypotheses about variation in competition will augment explanations about variation in cooperation.

### (e) Women's cooperation extends beyond childcare

Discussions of female cooperation in real-world settings have largely been confined to reproductive concerns—childcare and provisioning of children. However, women and girls cooperate across domains as broad-reaching as those of men and boys, including coalition formation, political, ceremonial and social institutions, and exchange and support networks ([112], in this issue). Although their participation may be less formalized than men's, the ethnographic evidence summarized by Kramer [112] suggests that we need to reconsider the notion that the scope for cooperation among women and girls is limited by reproductive constraints.

The strategies women employ to form support networks should reflect the demands of the activity they are engaging in. For example, Mattison and colleagues ([81], in this issue) proposed that cooperative networks centred around food preparation, which is primarily women's work, and farm equipment lending, primarily men's work, in matrilineal and patrilineal Mosuo communities were shaped more by the type of activity than by expected gender norms around cooperation. Furthermore, women and girls are adept at exchanging cooperation across domains to balance multiple needs, such as childcare and productive labour. Starkweather and colleagues ([122], in this issue) showed that the cooperative childcare and work partner networks of Shodagor women in rural Bangladesh differed according to women's economic activities. Women who cooperated together as traders were also likely to provide childcare for each other. Since trading is incompatible with childcare, this exchange allowed them to solve the tradeoff between allocating time to childcare and economic activities. By contrast, fishing is compatible with childcare, and women who worked as fishers had narrower allocare networks.

Considering that women's cooperative networks change with the task at hand and that women cooperate across diverse domains, more evidence-based ethnographic support is needed to capture the breadth of women's cooperative behaviour. Without this data, understanding the factors that shape women's cooperation across societies or making comparisons with men's cooperation will remain cursory.

## 4. Conclusion

The multi-disciplinary research in this issue situates women's cooperative behaviour in a larger, cross-cultural, evolutionary context. In doing so, contributions challenge the narrative of clear-cut, biologically rooted gender differences in cooperation and confront the current perspective that women's cooperation is rare. Authors repeatedly demonstrate that women's cooperative bonds extend beyond networks of close kin, push us to think about women's cooperation outside of childcare, and suggest that women's ability to flexibly leverage relationships with available partners is rooted in our evolutionary past. Additionally, research in this issue urges us to consider variables beyond marital residence patterns, access to kin and competition as determinants of the scope of women's and girl's cooperation. Authors break further ground by proposing novel hypotheses about how women's cooperation responds to risk, wealth accumulation, cultural norms and economic needs. Yet, we remain in need of broad, theoretical discussions of the conditions that promote and constrain women's behavioural strategies. Studies examining how women's cooperation is shaped by changes over their lifespans, including life-history transitions and demographic changes in access to cooperative partners, and how women's cooperation differs in between- and within-group contexts are sparse. With the accumulation of large-scale, long-term datasets, researchers should also endeavour to answer questions about the impact that cooperation has on women's health, survival and reproductive outcomes. These advances will be necessary to establish an improved, holistic model of human sociality that recognizes the importance of cooperation for both men and women, adults and children.

## Data Availability

This article has no additional data.
